# The Need for an Innovative, Affordable, and Quality Hemodialysis Device in India

**DOI:** 10.1016/j.ekir.2023.06.015

**Published:** 2023-06-29

**Authors:** Manjunath Shetty, Shyam Vasudev Rao, Kiran KK, Padmanabha Holla, Ravi Prakash Deshpande, Urmila Anandh

**Affiliations:** 1 Department of Nephrology, JSS Medical College, Mysuru, India; 2 Renalyx Health Systems, Bangalore, India; 3 Department of Nephrology, Amrita Hospitals, Faridabad NCR, India

## Introduction

With increasing life expectancy and prevalence of lifestyle diseases, India is experiencing a dramatic increase in the incidence of diabetes and hypertension. With this epidemiologic change, there is also a commensurate increase in the incidence and prevalence of end-stage kidney disease. For these vulnerable patients, hemodialysis (HD) improves survival and quality of life. Many centers worldwide are showing first year survival to be higher than 75%.^1^ In contrast, mortality still remains high in resource- limited world where socioeconomic factors have a major impact on patient survival.^2^ Most of these patients are unable to access and/or afford dialysis therapies. Despite efforts by the government, dialysis therapy is fraught with many obstacles. One of the major challenges is in scaling up of infrastructure, manpower, and machines, which are required to meet this burgeoning demand. In India, the lack of dialysis services and accessibility have a major impact on outcome.^3^ Of 200,000 people who develop end-stage kidney disease in India every year, only 25% are urban dwellers. The rest are from remote corners of the country who travel long distances to reach dialysis centers.^4^ As the number of patients increase, the strain on the virtually nonexistent HD services is likely to increase further. An estimated 80% of this incident Indian end-stage kidney disease population do not survive and the deaths have doubled over a 10-year period (2001–2003 and 2010–2013).^5^ India is slowly setting up a National Dialysis Program, which will further increase the demand of dialysis services.^6^ The dialysis equipment market world over, including India, is currently led by foreign multinational companies. Imported dialysis machines are associated with challenges of affordability, availability of spares, and lack of regular maintenance.

In addition, the cost of such massive exercise in providing affordable care to patients with end-stage kidney disease is prohibitive. Therefore, there is an urgent unmet need to provide domestically manufactured, innovative, cost-effective, high quality dialysis devices, consumables, and services to reduce prices, improve affordability and support sustainable access to therapy. To this end, utilization of home-grown technology and indigenously built HD machines will go a long way in reducing the expenditure. In this paper, we present the details of the development of a “make in India” hemodialysis machine, which attempts to address some of the pressing issues relevant to financially constrained and geographically vast country such as India. This device is a regulation compliant HD device prototype. It is enabled with remote connectivity and clinical data access, which will improve reach and quality of care.

## Design and Development

The design process and development life cycle of the HD device was initiated in 2014, with adherence to biomedical device design, and development lifecycle protocols. The circuit requirements were identified, subsystems developed, and the prototype industrial design was then manufactured, keeping the available conventional technology as the base. Deficiencies, needs, and glitches were comprehensively documented during the entire development process. Focusing on the patient and provider at all times, the process of device development emphasized the following core systems: biocompatibility, reliability, durability, and serviceability.

**Figure 1:**
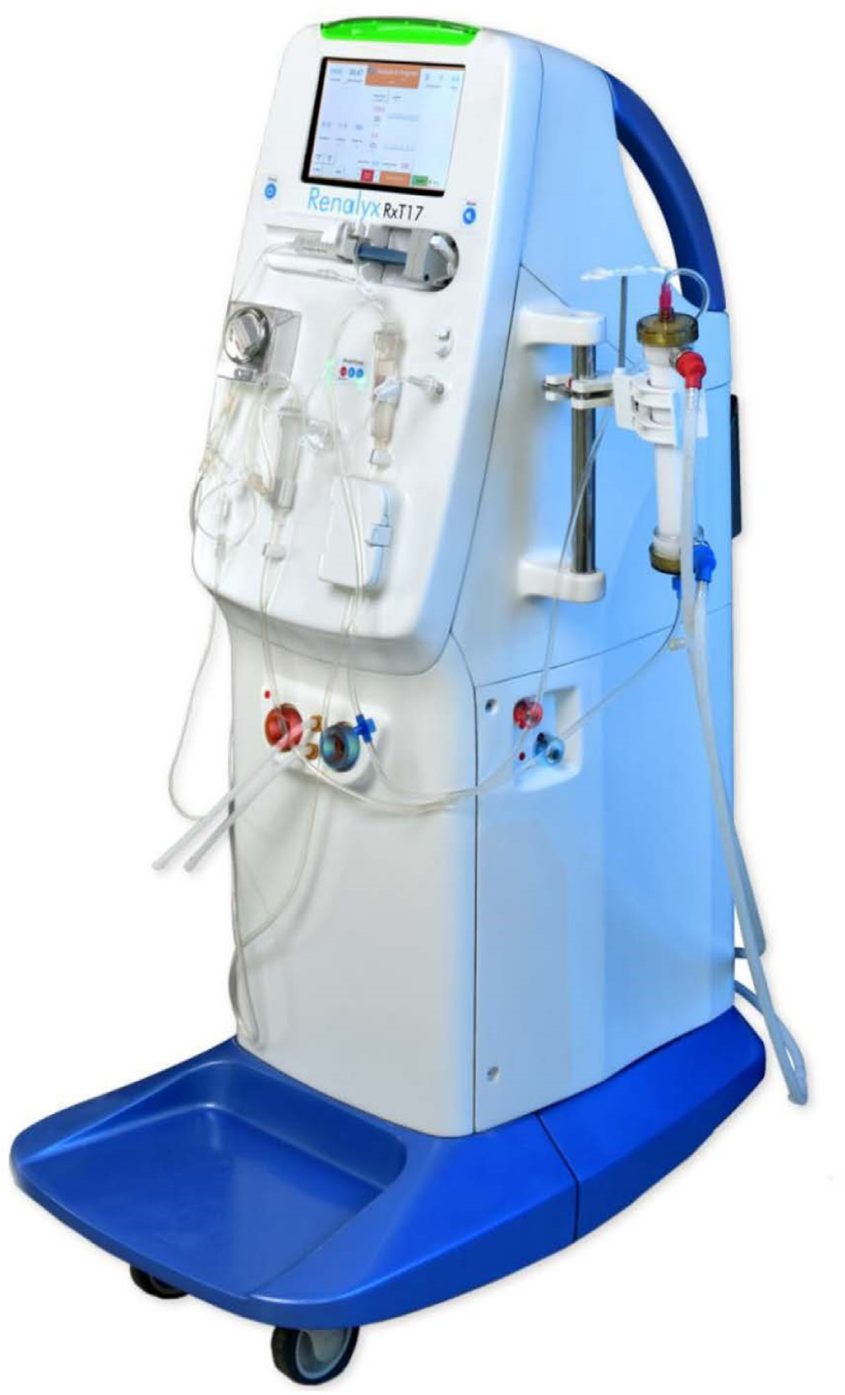
The prototype hemodialysis machine RxT17.

All the engineers involved were exposed to the hemodialysis unit and patients with the guidance of the clinicians involved, to obtain a clear understanding of the device’s functionality requirements and its clinical impact. Local and quality guided components of critical hydraulic (balancing chambers) and electronic components (e.g., sensors, valves) were developed. A dedicated expert facility for manufacturing was developed. It was decided that the device should include remote monitoring features, not only essential for rural outreach, but to provide tele-nephrology capabilities. There were many challenges encountered during the design and development of the HD machine. The difficulties and their solutions are summarized in Table 1. With all the above in mind, the prototype RxT17 (Figure 1) was developed. This machine is 80% indigenized.

**Table 1:** Challenges faced during development and corrective steps

Serial number (Sl.No.)	Challenges faced	Corrective steps
1.	Understanding the working principles and technology.	Numerous hours spent at the hospitals observing the dialysis procedures. Discussions with nephrologists and dialysis therapists. Study of various user and service manuals available on the web.
2.	Availability of components suitable for an invasive medical device, with biocompatibility.	Considering that the manufacture of medical devices in the country is nascent, sources for suitable components were identified. It was consciously decided to import components for the development of the machine, and then take up indigenization of the components.
3.	Finalizing the required specifications.	Discussions with physicians on the following: 1. Necessary features. 2. Shortfalls in existing machine. 3. Redundant features provided by machine manufacturers, whose design was based on the conditions of western patients. 4. Collecting Indian patient data.
4.	Limitations in availability of raw materials for various hydraulic parts.	Importing the suitable machinable plastic PEEK (at high cost) and subsequently replacing with PEI of equivalent one.
5.	Functional testing: 1. Usage of water during system testing (led to improper UF goals) 2. Adjusting Na+ limits within ±3 millimoles/l.	1. Replace water with expired blood in the extracorporeal circuit for simulated testing. (Done in very limited cases because expired blood hemolyzes very fast). By using reinforced silicone tubes, which avoided the expansion of plain silicone tubing because of pressures post-degassing pump. 2. Considerable testing and retuning of electronic circuits and software.
6.	Requirement of animal trials before clinical trials. (This led to time loss because of customization of machine to match animal weight and blood) Difficulty in conducting clinical trials in a completely new scenario in the country. Because the machine was developed for the first time in India, procedures for conducting clinical trials were difficult.	Renalyx had to work with regulatory authorities to develop a system for animal trials in a developing device. Considerable time was spent testing and gathering test data to prove suitability of the machine for human trials.
7.	Numerous hurdles in clearing the CE certification because of nonavailability of local experts who could help us with approval.	The machine had to be taken to different locations, even to Pune, for testing as per ISO 60601-1. −2, and −16 standards. The final clinical data had to be sent to Germany for expert review and clearance by TUV-SUD AG, Germany.
8.	Stage-wise development of electronics led to multiple numbers of PCBs and thereby reduced reliability.	The consolidation of PCB into lesser no of PCBs was taken up during second phase.
9.	Angle welded frames for the prototype had considerable distortion leading to assembly problems.	Frame design was modified using formed sheet metal sides with minimum no. of welded cross members.

PEI, poly ether imide; PEEK, poly ether ether keton; PCB, printed circuit board; TUV, Technischer Uberwachungsverein.

An open label, crossover, observational study was conducted with ethical approval and registration with the clinical trials registry of India (CTRI/2018/02/011882). The study was conducted successfully, and all patients completed the trial with no untoward or serious adverse events either during or between the sessions. The prototype machine was found to be noninferior to the available machines in the market (Supplementary Data S1).

Two units of RxT17 have been operational since 2019 at a medical college hospital in Mysuru in India. Because remote monitoring of the runtime, alarms, and other performance related parameters are built-in features, our engineers are able to keep track of such events.

Renalyx is Data Subject Access Request, Indian Standards Organization (ISO)13485 and Conformite Europeenne (CE) certified. The machine is Importer-Exporter Code (IEC) 60601-1 & IEC60601-1-16 compliant.

## Advantages of Renalyx Rxt17

**Figure 2:**
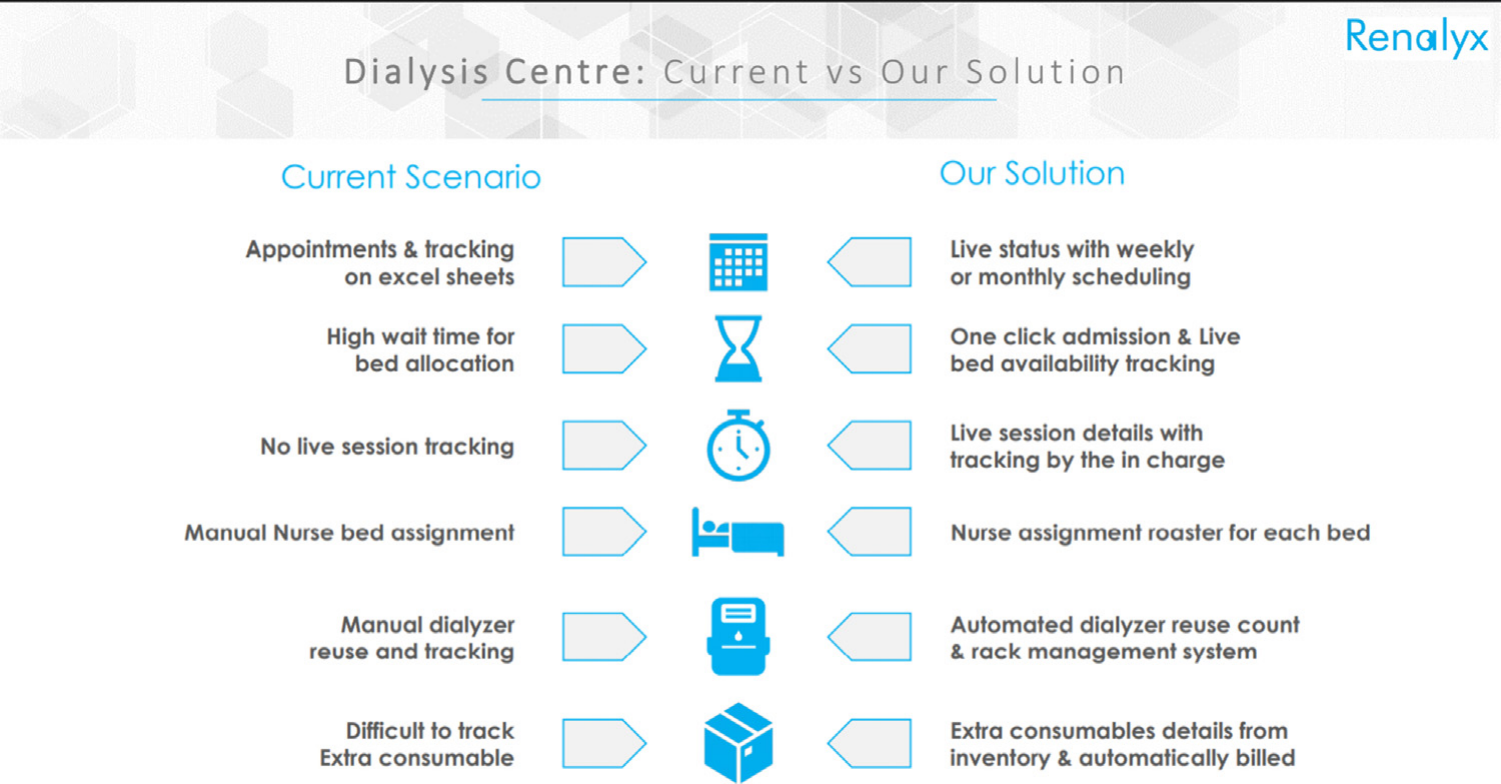
The key modules of the tele-nephrology platform.

The advantages of this machine are many; one of the obvious one is that it is indigenously developed, which reduces the economic “cost” of importing HD machines from the developed world. The machine is 20% cheaper than the regularly used machines. The cost will come down further when all components are indigenized. The usability of this device is also made simple by an on-screen user interface. The step-by-step instructions enable the nurse or technician to handle the machine effortlessly. The after-sales and service support is expected to be timely and the spares will be available as and when required. The remote monitoring feature and inbuilt tele-nephrology solutions are unique and are extremely useful when these machines are used in remote areas of the country and other low and middle-income countries with human resource constraints (Figure 2). The dialysis therapy data can also be evaluated with tele-nephrology. This will significantly improve not only the accessibility but also improve the efficiency of end-stage kidney care not only in India but in many low and middle-income countries. In India, many state governments are taking the lead in inducting “Make in India” equipment and we hope the machine, when in mass production, would be accepted in all corners of the country.

The machine systems are continuously being upgraded with endotoxin filters, single needle, and slow low efficiency dialysis capabilities. Simultaneously, all the spare parts are getting indigenized. The development of the HD machine has set in many nonrenal economic spinoffs for our country. The advantages of this machine for India and future developments are summarized in Table 2.

**Table 2:** The features of the machine and their advantage in dialysis delivery in India

Feature	Renalyx RxT17	Advantages
Machine details	Lighter weight (approx. 10 kg lighter than commonly used machines in India) Increased mobility 30%–40% cheaper.	Useful in LMICs where the machine can be easily moved from one location to another depending on the need. Significant cost reduction is a major advantage.
Service and parts supply	Engineers trained onsite. Most parts are indigenous in nature and/or are getting indigenized.	Improved utilization of the machine time. Significant reduction in recurrent expenses.
Monitor	Computerized programmable. Touch display.	The touch display is user friendly and can be used by staff in any part of the country.
Dialysate flow rate	300/500/700 ml An option of lower flow rate for SLED is being developed.	The lower upper limit of dialysate flow developed because 800 ml/min flow rate is often not used in India and leads to excessive water wastage. The SLED function in the same machine will facilitate use of the machine in ICUs.
Cleaning and disinfection	Water rinse, hot water rinse, bleach disinfection, hot citric acid disinfection.	Independence from captive disinfection materials from multinational companies. Large savings.
Single needle treatment function	Currently being developed.	Will be of immense benefit in LMICs such as India where skilled and trained manpower is scarce in remote and inaccessible parts of the country.
Technical data management system	Internet of Things (IoT) enabled machine.	Machine therapy data can be accessed and monitored remotely. Servicing of machines, diagnosis of malfunction can be done remotely, again addressing skilled technicians’ shortage in remote areas.
Inbuilt water treatment facility	Currently unavailable, in advanced stage of design and development.	Will address one of the major concerns of dialysis water purity. Will also reduce initial cost of setting up a dialysis unit because the installation of the water treatment plant makes up for the major chunk of expenditure.

ICU, intensive care unit; LMICs, low and middle-income countries; SLED, slow low efficiency dialysis.

## Discussion

The inequities that beset CKD patients in India are further magnified when they need renal replacement therapy. Not only socioeconomic deprivation but also accessibility to therapy are major barriers to care.^7^ Even when dialysis is offered free, they still dropout because traveling to far off dialysis units lead to loss of their daily wages.^8^

Even with governmental support,^6^ most patients cannot be accommodated within the existing hemodialysis infrastructure in India because of the prohibitive cost.^9^ To improve this situation, it would seem logical to develop an indigenous machine, which has been the focus of Indian engineering industry over the last few years.

Even though the device developed is regulatory compliant, much more needs to be done. In future, the manufacturers aim to scale the safety parameters and features of the device. The added features which will be incorporated will be the ability to perform slow dialysis and an inbuilt water treatment facility.

### Conclusion

The development of an indigenous low-cost HD device will advance cost-effective HD care delivery in India; and enable production of quality driven, cost-effective, and customizable devices in the future. It is envisaged that the commercial models would provide access to affordable and safe care and would help build an ecosystem for the manufacturing of dialysis-related components in India.

## Disclosure

All the authors declared no competing interests.

## Funding

The development of device in the initial stages received no funding. For the clinical trial, there was partial funding from Department of Science and Technology, Government of India, India Innovation Growth Program (IIGP 2.0).

## Supplementary Material

Supplementary File (PDF)

**Data S1.** Randomized Crossover Trial Data.
