# Supplementary material for: The Need for an Innovative, Affordable, and Quality Hemodialysis Device in India
Source: Kidney Int Rep. 2023 Jun 29;8(9):1879–82. doi: 10.1016/j.ekir.2023.06.015 (PMC10496065; doi:10.1016/j.ekir.2023.06.015)
Supplement: Supplementary File (PDF) [file mmc1.pdf]

**Clinical Trial Data** :

An open label, crossover, observational study was conducted with ethical approval and registration with the clinical trials registry of India [CTRI/2018/02/011882] . The study patients included five stable male patients (mean age 38± 8 years). The cause of their kidney disease was hypertensive nephropathy. The Fresenius 4008S dialysis machine provided for control. A total of 20 sessions were carried out (5 sessions on the Fresenius 4008S machine and 15 sessions on RxT17 HD device).(Figure 2) Patient characteristics (vitals, adverse events) and machine details (conductivity, trans-membrane pressure, temperature, ultrafiltration volume, alarms) were documented. Biochemical, microbiological, and haematological samples of the patients were collected and analysed. Any adverse events and device deficiencies were noted. A validated patient renal treatment satisfaction questionnaire (RTSQ) was used to understand therapy related concerns. All patients completed the trial with no untoward or serious adverse events either during or between the sessions. The analysis of the interclass coefficients of the laboratory variables is given Table.

The mean age patients were 38yrs±8yrs. All patients had stable vital signs with no serious adverse events during the dialysis sessions. All machine data measured were within the acceptable range of conductivity (± 3 mEq/L}, temperature (± 0.5 degree centigrade) and set ultrafiltration (UF) volume (within 10%} of target value. The mean± s.d. of KT/V (Urea) for Renalyx RxT17 was 1.15±0.3 and 0.96±0.5 for Fresenius 4008S. The Urea Reduction Ratio (%) was 56.7±12.3 for RxT17 and 49.0±19.1 for Fresenius 4008S. All the above values revealed a statistical conformity of the Renalyx Rx17 in comparison to the Fresenius 4008S machine.

Two subjects experienced muscle cramps (one each with the Fresenius and RxT17 dialysis machines) towards the end of the session. The cramps resolved after stopping the ultrafiltration (UF). lntradialytic hypotension was observed in a total of 3 HD sessions (1

session of Fresenius and 2 sessions of RxT17 ); here the UF was terminated and saline was infused to correct hypotension in each of the haemodialysis sessions. No mechanical cause of hemolysis was observed (LDH values). No microbiological growth in the blood was observed in cultures drawn following each dialysis session. All the patients responded to the RTSQ .They indicated that they were very satisfied with their therapy during the trial. One subject showed dissatisfaction with the session timing of the trial, while another subject found the standard HD interfered in his daily activities. The conclusion of this clinical trial indicated that the device RxT17 HD machine was comparable to Fresenius 4008S in efficacy and safety in delivering dialysis therapy to patients with end stage kidney disease.

**Table**

Table :Intraclass coefficient(ICC) of the laboratory values

| **FRESENIUS VS RENALYX** | **ICC** | **95%C.I** | |
|---|---|---|---|
| | | **LOWER** | **UPPER** |
| **Kt/V** | 0.95 | 0.58 | 0.99 |
| **URR%** | 0.94 | 0.46 | 0.99 |
| **LDH** | 0.91 | 0.19 | 0.99 |

URR- Urea Reduction Ratio; LOH- Lactate Dehydrogenase; ICC- Intra-class correlation coefficient
